# The Location and Appearance of Second Malignancies in Patients With Bilateral Retinoblastoma

**DOI:** 10.1080/13577149778353

**Published:** 1997-06

**Authors:** Carole Z. Rubin, Nancy S. Rosenfield, Sara J. Abramson, David H. Abramson, Ira J. Dunkel

**Affiliations:** 1 Department of Radiology Memorial Sloan Kettering Cancer Center 1275 York Avenue New York NY 10021 USA; 2 Department of Pediatrics Memorial Sloan Kettering Cancer Center and New York Hospital USA; 3 Department of Ophthalmology Memorial Sloan Kettering Cancer Center and New York Hospital USA

## Abstract

*Purpose*. This paper describes the clinical history and radiographic appearance of second malignancies in patients with bilateral retinoblastoma.

*Subjects/methods*. The imaging studies and clinical data of 14 patients with a history of bilateral retinoblastoma who were treated for second malignancies were reviewed.

*Results*. A total of 17 tumors were identified in 14 patients during the period 1978–1996. The median age of occurrence of the second malignancy was 17 years (range 10–32 years). Fourteen of the 17 malignancies occurred in the facial structures and three developed in the lower extremities. The histologies included osteosarcoma (*n* = 5), malignant fibrous histiocytoma (*n* = 3), high-grade spindle cell sarcoma (*n* = 3), malignant mesenchymoma (*n* = 1), leiomyosarcoma (*n* = 4) and angiosarcoma (*n* = 1). The tumors were locally aggressive and had a similar appearance to those found in nonretinoblastoma patients. Six of the 14 patients are alive and disease free.

*Discussion*. Most of the adolescent and young adult retinoblastoma survivors developed second malignancies in the irradiated facial structures but some occurred in distal sites. Radiologically, these tumors do not differ in appearance from those seen in non-retinoblastoma patients with the exception of their location.


*Sarcoma* (1997) 1, 89–93

ORIGINAL ARTICLE

### The location and appearance of second malignancies in patients with bilateral retinoblastoma

CAROLE Z. RUBIN,[1] NANCY S. ROSENFIELD,[1] SARA J. ABRAMSON,[1] DAVID H. ABRAMSON[3] & IRA J. DUNKEL[2]

*Departments of [1]Radiology, [2]Pediatrics and [3]Ophthalmology, Memorial Sloan Kettering Cancer Center and New York Hospital, USA*

**Abstract**
*Purpose.* This paper describes the clinical history and radiographic appearance of second malignancies in patients with bilateral retinoblastoma.
*Subjects/methods.* The imaging studies and clinical data of 14 patients with a history of bilateral retinoblastoma who were treated for second malignancies were reviewed.
*Results.* A total of 17 tumors were identified in 14 patients during the period 1978–1996. The median age of occurrence of the second malignancy was 17 years (range 10–32 years). Fourteen of the 17 malignancies occurred in the facial structures and three developed in the lower extremities. The histologies included osteosarcoma ($n = 5$), malignant fibrous histiocytoma ($n = 3$), high-grade spindle cell sarcoma ($n = 3$), malignant mesenchymoma ($n = 1$), leiomyosarcoma ($n = 4$) and angiosarcoma ($n = 1$). The tumors were locally aggressive and had a similar appearance to those found in non-retinoblastoma patients. Six of the 14 patients are alive and disease free.
*Discussion.* Most of the adolescent and young adult retinoblastoma survivors developed second malignancies in the irradiated facial structures but some occurred in distal sites. Radiologically, these tumors do not differ in appearance from those seen in non-retinoblastoma patients with the exception of their location.

**Key words:** *retinoblastoma, second malignancies, clinical features.*

### Introduction

Retinoblastoma (RB) is a rare ocular neoplasm of childhood which occurs in a sporadic or hereditary form. While the sporadic form typically presents with unilateral disease, most patients with the hereditary form have bilateral retinoblastoma (BRB). The hereditary form is associated with a germ-line defect in the retinoblastoma gene, RB-1, on chromosome 13 and is transmitted in an autosomal dominant pattern. Prognosis for patients with BRB is excellent with approximately 90% of patients achieving cure of the primary disease.[1,2] However, these same patients are at an increased risk of developing a second malignancy (SM).[3–5] This paper summarizes the experience at our institution regarding second malignancies in patients treated for BRB and describes the radiographic features.

### Subjects and methods

A series of 14 patients with BRB presenting with a SM was obtained via a search of the Memorial Hospital database. Retrospective analysis of the charts, pathology and radiologic studies were performed.

### Results

A total of 14 patients with BRB were treated for a second malignancy at our institution during the period 1978–1996. Tables 1 and 2 summarize the clinical history and outcome of these 14 patients.

Thirteen of the patients were diagnosed with BRB by 21 months of age. One patient (12) is included in the series because his unilateral RB was multi-focal and he has a family history of RB. Each patient initially underwent enucleation of one globe and most patients had radiation therapy given to the opposite eye. Two of the patients had radiation therapy to both eyes. Initial radiation therapy doses ranged from 3575 cGy to 5400 cGy. The radiation doses for six of the patients could not be ascertained as their radiation treatment was performed elsewhere. The age at diagnosis of the SM ranged from 10 to 32 years with a median age of 17 years.

Correspondence to: N. S. Rosenfield, Department of Radiology, Memorial Sloan Kettering Cancer Center, 1275 York Avenue, New York, NY 10021, USA. Tel: +1 212 639 5512; Fax: +1 212 794 4010; E-mail: nrosenfield@medim.mskcc.org.

1357-714X/97/020089-05 $9.00 ©1997 Carfax Publishing Ltd

**Table 1.** *Clinical history of nine patients treated for SM after RB*

| Patient | Age at diagnosis of BRB (months) | Treatment of BRB | Age at SM (years) | Site of SM | SM in radiation port |
|---|---|---|---|---|---|
| 1 | < 12 | Enucleation OD<br>RT OS: dose? | 19 | Left ethmoid | Probable |
| 2 | 13 | Enucleation OD<br>RT OS: 3575 cGy | 14 | Left orbit | Yes |
| 3 | 8 | Enucleation OS<br>RT OD: 6900 cGy | 10 | Right maxilla | Yes |
| 4 | 18 | Enucleation OS<br>RT OS: dose? | 19 | Left orbit | Probable |
| 5 | 6 | Enucleation OS<br>RT OD: dose?<br>Enucleation OD | 11 | Right orbit | Probable |
| 6 | 3 | Enucleation OD<br>RT OS: 3500 cGy | 16<br>18 | Left tibia<br>Right orbit | No<br>Yes |
| 7 | 4 | Enucleation OD<br>RT OU: 4500 cGy | 13 | Left maxilla | Yes |
| 8 | 9 | Enucleation OD<br>RT OS: 5400 cGy | 25 | Left orbit | Yes |
| 9 | 11 | Enucleation OS<br>RT OU: 4500 cGy | 18 | Left malar | Yes |
| 10 | 18 | Enucleation OS<br>RT 4500: 5000 cGy | 12<br>14 | Right ethmoid<br>Upper lip | Yes |
| 11 | 19 | Enucleation OS<br>RT | 13<br>23 | Right femur<br>Left sphenoid | No<br>Probable |
| 12 | 5 | RT: 3648 cGy | 32 | Right ethmoid | Yes |
| 13 | 18 | Enucleation OD<br>RT OS | 20 | Left femur | No |
| 14 | 21 | Enucleation OD<br>RT OS: 4500 cGy<br>Enucleation OS | 22 | Left thigh | No |

SM = second malignancy; RB = retinonblastoma; BRB = bilateral retinoblastoma; RT = radiation therapy; OD = right bone; OS = left globe; OU = both globes.

Patient 6 was diagnosed with a SM at 16 years of age, and then developed a third malignancy at age 18 years . This third malignancy, malignant fibrous histiocytoma (MFH) in the right orbit, was different in location and histology than the second malignancy, an osteosarcoma of the left tibia.

Patient 10 developed a third malignancy, angiosarcoma of the upper lip, 16 months after radiation therapy for leiomyosarcoma of the right ethmoid sinus.

Patient 11 developed an osteosarcoma of the femur 13 years after treatment for BRB and a leiomyosarcoma within the facial radiation field 22 years after irradiation.

The histologies of the malignancies included five osteosarcomas (OS), three high-grade spindle cell sarcomas (HGS), one angiosarcoma, one malignant mesenchymoma, three malignant fibrous histiocytomas (MFN) and four leiomyosarcomas (LMS). Fourteen of the 17 SM occurred within the facial structures; six known to be within the radiation port. In six patients, it is highly likely that the SM occurred in the radiation port. One patient whose SM was not in the facial bones ultimately developed a third malignancy within the radiated facial structures.

Radiographically, the OS in these patients had a similar appearance to those in non-BRB patients. The tumors demonstrated a combination of osteolysis and osteosclerosis and an associated soft tissue mass. Osteoid matrix was also present (Fig. 1). Enhancement of these lesions was present on the contrast-enhanced computed tomography (CT) and magnetic resonance imaging (MRI) studies. The location of the OS, however, was unusual. One of the five OS developed in the long bones (the common location for *de novo* OS) while the other four developed in the facial bones.

The MFH were heterogeneous-enhancing lesions which caused osseous destruction which was

**Table 2.** *Outcome of nine patients with SM after RB*

| Patient | Site of SM | Pathology of SM | Treatment of SM | | Clinical outcome (months) |
|---|---|---|---|---|---|
| 1 | Left ethmoid | OS | Chemotherapy | Surgical resection | EFS: 91 |
| 2 | Left orbit | OS | Chemotherapy | | D: 27 |
| 3 | Right maxilla | LMS | Chemotherapy | Radiation therapy | D: 16 |
| 4 | Left orbit | HGS | Partial resection | Chemotherapy | ST: 59 |
| 5 | Right orbit | MFH | Chemotherapy<br>Radiation therapy | Surgical resection | D: 73 |
| 6 | Left tibia<br>Right orbit | OS<br>MFH | Chemotherapy<br>Chemotherapy | Surgical resection<br>Surgical resection | D: 35 |
| 7 | Left maxilla | OS | Chemotherapy | Surgical resection | EFS: 214 |
| 8 | Left orbit | MFH | Chemotherapy | Surgical resection | D: 98 |
| 9 | Left malar | HGS | Chemotherapy | Surgical resection | EFS: 102 |
| 10 | Right ethmoid<br>Upper lip | LMS<br>Angiosarcoma | Surgical resection<br>Surgical resection | Radiation therapy | 24 + |
| 11 | Right femur<br>Left sphenoid | OS<br>LMS | Chemotherapy<br>Radiation therapy | Surgical resection<br>Chemotherapy | 148 + |
| 12 | Right ethmoid | LMS | Surgical resection | Radiation therapy | 6 + |
| 13 | Left femur | HGS | Surgical resection | | 25 + |
| 14 | Left thigh | Mesenchymoma | Surgical resection | Radiation therapy | 24 |

SM = second malignancy; RB = retinonblastoma; OS = osteosarcoma; LMS = leiomyosarcoma; HGS = high-grade spindle cell sarcoma; MFH = malignant fibrous histiocytoma; EFS = event-free survival; D = decreased; ST = surviving with tumor.

typically permeative. Like the OS, the MFH had an appearance similar to that of a *de novo* MFH but the location was unusual.

The three cases of HGS appeared as inhomogeneous masses with central necrosis and contrast enhancement on MRI and CT. The tumors caused destruction of the adjacent osseous structures but no matrix was seen in the soft tissue component. In one case, patient 4, the HGS started in the left orbit and caused proptosis of the left globe.

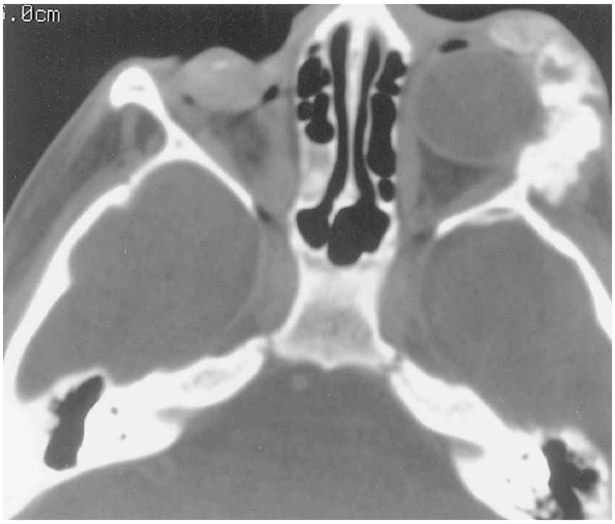

**Fig. 1.** *Patient 2, a 14-year-old female, with osteosarcoma of the left orbit with osseous matrix formation on axial CT. The right globe has been enucleated.*

Four patients developed a LMS in our group. This heterogeneous soft tissue tumor infiltrated the paranasal sinuses, and sometimes extended to the orbit and extended intracranially to destroy the skull base.

Intracranial extension of the tumor was not uncommon. Three had intracranial tumor at the time of presentation; patient 5 with MFH, patient 4 with HGS (Fig. 2) and patient 3 with LMS. Patient 2 may have had intracranial extension of the OS but the imaging studies performed prior to treatment were equivocal. Only one of these four patients, patient 5, went on to have a complete surgical resection.

While the tumors were frequently large at the time of presentation, none had metastatic disease at the time of their initial work-ups. Later in their disease, patients 3, 5 and 14 developed thoracic metastases and patient 11 developed abdominal metastatic nodules. Patient 3, initially diagnosed with LMS of the right maxilla, developed pulmonary nodules. Forty-four months after treatment of the MFH of the right orbit, patient 5 had metastatic MFH to the right pleural space (Fig. 3) and later intrapulmonary disease.

Nine of the 14 patients in this study experienced recurrence of their tumors.

Other radiographic features common to these patients are associated with the therapy they received

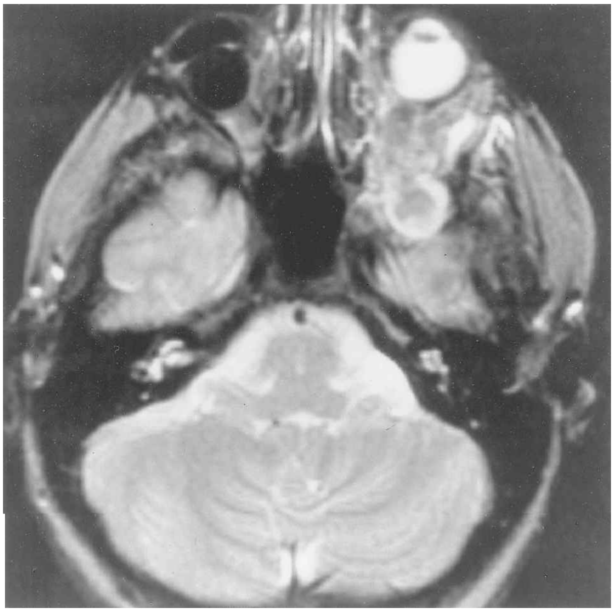

**Fig. 2.** *High-grade spindle cell sarcoma of the left orbit extending into the middle cranial fossa on an axial T2 weighted (2500/85) MRI image of patient 4, a 19-year-old female. A prosthesis is present in the right orbit.*

as treatment for their BRB or SM. Hypotelorism resulting from radiation-induced bone injury to the orbit and adjacent bony structures was seen in several patients. Cerebral white matter changes from radiation and chemotherapy were visible on MRI.

Six of the 14 patients have died of causes related to their malignancies, after surviving a median of 31 months from diagnosis of the SM. Eight patients are alive, of which six are disease free at a median of 58 months after the SM was diagnosed. Two others are alive with residual disease.

### Discussion

It has been recognized that patients with BRB are at greater risk of developing a SM. Abramson *et al.* determined that two-thirds of the second tumors arise within the field of radiation therapy and one-third are outside the field.[2] This suggests that radiation therapy used to treat retinoblastoma may play a part in the induction of the SM but there must also be a genetic predisposition. Genetic studies have demonstrated that the RB-1 gene whose deletion is responsible for retinoblastoma is localized on band 14 of the long arm of chromosome 13. RB-1 mutations have also been identified in OS.[6–8]

In our study, all the patients received radiation therapy as part of their treatment for BRB and developed a sarcoma within the field of radiation. As with radiation-induced sarcomas in non-RB patients, the sarcomas developed after a long latency period. Our patients were diagnosed and treated for BRB in infancy. The sarcomas which they developed occurred one to three decades later at a median age of 17 years with a range of 10–32 years.

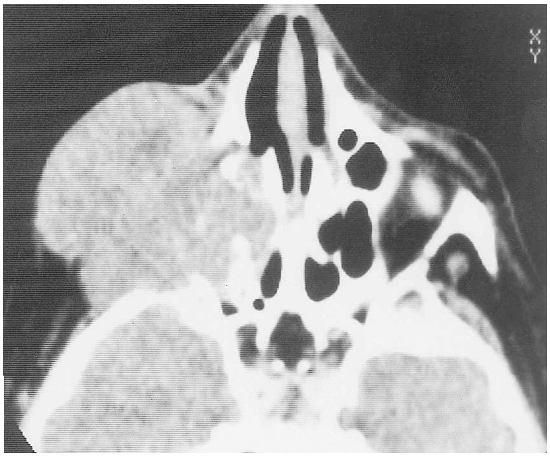

(a)

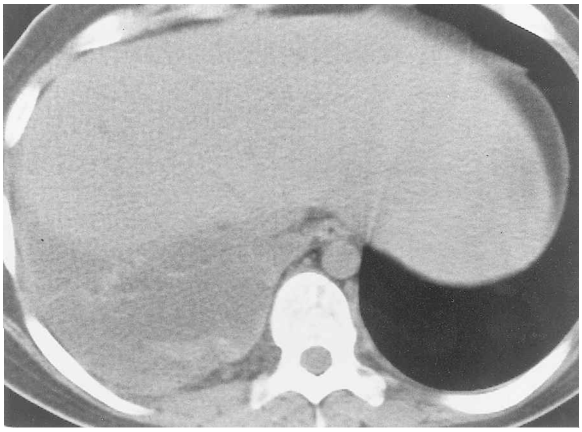

(b)

**Fig. 3.** *Malignant fibrous histiocytoma involving the right orbit and right pleural space in patient 5, an 11-year-old male. (a) Heterogeneous soft tissue mass in right orbit causing destruction of the right ethmoid and right maxilla on coronal CT scan. (b) Axial CT image of the thorax demonstrating a soft tissue mass in the right pleural space.*

Radiation-induced sarcomas in non-RB patients have been reported to develop 4–30 years after completion of radiation therapy.[10]

The doses of radiation used to treat our patients were in the range which can result in osseous damage. Doses greater than 3000 cGy usually cause permanent damage to reparative mechanisms while doses greater than 5000 cGy can cause devitalization of bones.[10] These numbers were determined for adult bones; children may more favorably tolerate higher doses. Kin *et al.* reviewed the radiation-induced sarcomas of bone following therapeutic radiation at the Memorial Sloan Kettering Cancer Center from 1977 to 1982.[11] The age of the 10 patients in his study ranged from 9 to 52 years with five patients under 18 years of age. He found both osseous and extraosseous sarcomas can develop after receiving doses ranging from 3000 to 6000 cGy, with a median dose of 4605 cGy.

With the exception of their location, the radiographic appearance of the sarcomas in our patients was similar to those in non-BRB patients. In our study, only two of the five OS developed in a long bone. Typically, OS arise in the metaphyseal region of the tubular bones with 50–75% developing around the knee. Only 5–10% of *de novo* OS develop in the flat bones[12] yet three of the five OS in our patients developed there.

The tumors were locally advanced at the time of their presentation and had a propensity to recur, which may account for the poor clinical outcome for some of these patients. At our institution, aggressive treatment for the SM is advocated. Neo-adjuvant chemotherapy produced responses in the majority of the patients treated and, with subsequent surgery, long periods of disease-free survival were obtained. Six of our 14 patients are alive without evidence of disease, indicating that apparent cures can be achieved in a significant minority.

**Acknowledgements**

We would like to acknowledge Dr Fereshteh Ghavimi for her inspiration and guidance with this project, and Mrs Patricia Dudley and Ms Lennora Spicer who prepared the manuscript.

**References**

1 Abramson DH, Ellsworth RM. The surgical management of retinoblastoma. *Ophthalmic Surg* 1980; 11:596–8.
2 Abramson DH, Ellsworth RM, Kitchin FD, *et al.* Second nonocular tumors in retinoblastoma survivors. *Ophthalmology* 1984; 91:1351–5.
3 Hawkins MM, Draper GJ, Kingston JE. Incidence of second primary tumours among childhood cancer survivors. *Br J Cancer* 1987; 56:339–47.
4 Kingston JE, Hawkins MM, Draper GJ, *et al.* Patterns of multiple primary tumours in patients treated for cancer during childhood. *Br J Cancer* 1987; 56:331–8.
5 Eng C, Li FP, Abramson DH, *et al.* Mortality from second-tumors among long-term survivors of retinoblastoma. *J Natl Cancer Inst* 1993; 85:1121–8.
6 Lele KP, Penrose LS, Stallard HB. Chromosome deletion in a case of retinoblastoma. *Ann Hum Genet* 1963; 27:171–4.
7 Knudson AG Jr, Meadows AT, Nichols WW, *et al.* Chromosomal deletion and retinoblastoma. *N Engl J Med* 1976; 295:1120–3.
8 Yunis JJ, Ramsay N. Retinoblastoma and sub band deletion of chromosome 13. *Am J Dis Child* 1978; 132:161–3.
9 Hansen MF, Koufos A, Gallie B, *et al.* Osteosarcoma and retinoblastoma: a chromosomal mechanism revealing predisposition. *Proc Natl Acad Sci* 1985; 82:6216–20.
10 Wilner D. *Radiology of bone tumors and allied disorders.* Philadelphia: Saunders 1982; 2015–24, 3184–5.
11 Kin J H, Chu FB, Woodward HQ, *et al.* Radiation-induced sarcomas of bone following therapeutic radiation. *Int J Radiat Oncol Biol Phys* 1983; 9:107–10.
12 Resnick D, Niwayama G. *Diagnosis of bone and joint disorders.* 2nd edn. Philadelphia: Saunders, 1988; 3649.
13 Sagerman RH, Cassady JR, Tretter P, *et al.* Radiation induced neoplasia following external beam therapy for children with retinoblastoma. *Am J Roentgen* 1969; 105:529–35.
